# An interview with Hyeon-Shik Hwang

**DOI:** 10.1590/2177-6709.21.1.024-033.int

**Published:** 2016

**Authors:** Guilherme Thiesen, Telma Martins de Araújo, Maria Perpétua Mota Freitas, Alexandre Trindade Simões da Motta

**Affiliations:** »Professor, Universidade do Sul de Santa Catarina (UNISUL), Department of Orthodontics, Florianópolis, Santa Catarina, Brazil. » PhD in Dentistry, Universidade Luterana do Brasil (ULBRA), Canoas, Rio Grande do Sul, Brazil. » MSc in Orthodontics and Facial Orthopedics, Pontifícia Universidade Católica do Rio Grande do Sul (PUCRS), Porto Alegre, Rio Grande do Sul, Brazil. » Diplomate by the Brazilian Board of Orthodontics and Dentofacial Orthopedics (BBO).; »Full professor, Universidade Federal da Bahia (UFBA), Department of Orthodontics, Salvador, Bahia, Brazil. » Coordinator of UFBA José Édimo Soares Center of Orthodontics and Facial Orthopedics, Salvador, Bahia, Brazil. » MSc and PhD in Orthodontics, Universidade Federal do Rio de Janeiro (UFRJ), Rio de Janeiro, Rio de Janeiro, Brazil. » Associate editor, Dental Press Journal of Orthodontics. » Former chairman of the Brazilian Board of Orthodontics and Facial Orthopedics (BBO).; »Adjunct professor, Universidade Luterana do Brasil (ULBRA), Canoas, Rio Grande do Sul, Brazil. » PhD in Dentistry, Pontifícia Universidade Católica do Rio Grande do Sul (PUCRS), Porto Alegre, Rio Grande do Sul, Brazil. » MSc in Orthodontics and Facial Orthopedics, Pontifícia Universidade Católica do Rio Grande do Sul (PUCRS), Porto Alegre, Rio Grande do Sul, Brazil. » Scientific editor of Stomatos Journal.; »Adjunct professor, Universidade Federal Fluminense (UFF), School of Dentistry, Niterói, Rio de Janeiro, Brazil. » Chairman of the graduate program in Orthodontics, Universidade Federal Fluminense (UFF), School of Dentistry, Niterói, Rio de Janeiro, Brazil. » Specialist, MSc and PhD in Orthodontics, Universidade do Estado do Rio de Janeiro (UERJ), Rio de Janeiro, Rio de Janeiro, Brazil. » Research Fellow - University of North Carolina.

## Abstract

It gives me great pleasure to interview Dr. Hyeon-Shik Hwang, an innovative orthodontist who has developed many creative techniques over his career. Dr. Hwang was born in Korea and received his DDS and PhD degrees from Yonsei University in Seoul. He is professor and chairman of the Department of Orthodontics at Chonnam National University School of Dentistry, Gwangju, Korea. Dr. Hwang, as a faculty at the university hospital, has maintained a successful clinical practice for more than 25 years. He has treated many adult patients focusing on esthetics and periodontal health and has developed many clinical techniques to improve the effectiveness and efficiency of treatment to the benefit of both the patient and practitioner. Dr. Hwang is also interested in the evaluation of facial asymmetry two- and three-dimensionally. As one of the early adopters of cone-beam volume imaging, he has given special emphasis on the management of surgical cases. He is married to Jung-Un Park with whom he has two sons. His favorite hobbies are photography and listening to music. When I was presented to him in a congress, it was a great pleasure meeting someone who I already admired for his singular work. Later on, his humbleness and knowledge made me marvel at him even more. I hope that all readers of Dental Press Journal of Orthodontics also enjoy the teachings from this brilliant Korean orthodontist! Guilherme Thiesen - interview coordinator

## Abstract (pt)

É uma grande satisfação poder entrevistar, pelo *Dental Press Journal of Orthodontics* , o Dr. Hyeon-Shik Hwang, um ortodontista inovador que tem desenvolvido muitas técnicas criativas ao longo de sua carreira. Dr. Hwang nasceu na Coreia do Sul e recebeu seus títulos de DDS e PhD pela *Yonsei University* , em Seul. Ele é professor e chefe do departamento de Ortodontia da Faculdade de Odontologia da *Chonnam National University* , em Gwangju, Coreia do Sul. Dr. Hwang é membro do corpo docente do hospital universitário, e tem mantido uma prática clínica bem-sucedida por mais de 25 anos. Ele tem tratado muitos pacientes adultos, com foco na estética e saúde periodontal, e desenvolveu técnicas clínicas para melhorar a eficácia e eficiência do tratamento, beneficiando tanto pacientes quanto profissionais. Dr. Hwang tem interesse científico especial na avaliação bidimensional e tridimensional de assimetrias faciais. Como um dos pioneiros a adotar a tomografia volumétrica de feixe cônico, acabou dando ênfase especial para o tratamento de casos cirúrgicos. Ele é casado com Jung-Un Park, com quem tem dois filhos. Seus *hobbies* favoritos são a fotografia e a música. Quando fui apresentado a ele em um congresso, foi um grande prazer conhecer alguém que eu já respeitava, por seu trabalho singular. Mais tarde, sua humildade e inteligência me fizeram admirá-lo ainda mais. Espero que todos os leitores do *Dental Press Journal of Orthodontics* também possam desfrutar dos ensinamentos desse brilhante ortodontista coreano! Guilherme Thiesen - coordenador da entrevista

**Figure:**
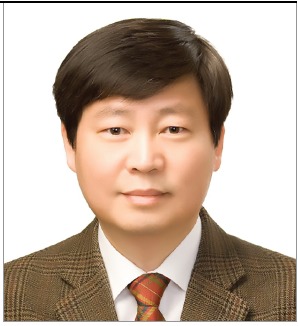

## Your knowledge about craniofacial asymmetries is singular, and your papers on 2D and 3D evaluation of these deformities are classic. How much do you think CBCT images have contributed to the diagnosis of dentofacial asymmetry? What was not possible to be properly analyzed in the past, with two-dimensional images? (Guilherme Thiesen)

Needless to say, CBCT has contributed very much to the diagnosis of dentofacial asymmetry. One of the main advantages of CBCT in the diagnosis of facial asymmetry is that frontal cephalograms can be generated in a standardized and reproducible manner.^1^^,^ 2 Although 3D image is available, it is believed that frontal cephalograms are better than 3D image for determining the presence and the degree of facial asymmetry. However, conventional radiographs have the inherent limitations of enlarging and distorting the image, and this can lead to misdiagnosis. It is because all cephalograms are obtained in perspective projection. In contrast, cephalograms can be generated in 'parallel' projection using CBCT volume image. Clear and accurate cephalograms without distortion and/or enlargement can be generated.^3^ We call this CG ceph (CBCT-generated cephalograms). In addition to accurate diagnosis, this imaging allows superimposition of frontal cephalograms to be obtained. In the past, we had difficulty with the superimposition of frontal cephalograms due to a difference in head posture. Using computer algorithm, reorientation of second volume image into the same position, as in the initial volume image, became possible. As a result, two CG cephs can be generated in the same posture and angle. Standardized and reproducible cephalograms can be generated simply from CBCT volume images. Not only accurate diagnosis, but also precise treatment evaluation became possible with the help of this unique imaging modality, CG ceph. 

## How do you see the current status of CBCT use in orthodontic diagnosis and treatment planning by Asian clinicians as well as around the world? Do you think that published guidelines and clinical recommendations from the European SEDENTEXCT and the American Academy of Oral and Maxillofacial Radiology, as well as routine aspects like software and exam costs, the time consumed in diagnosis and even lack of training, have been influencing the way orthodontists deal with 3D imaging? (Alexandre Trindade Simões da Motta)

Whether routine use of CBCT in orthodontic diagnosis and treatment planning is justified is one of the hottest issues in today's Orthodontics. If somebody asks me about it, my answer would be: "It depends on the practitioner." Many other clinicians will say it depends on the "case." But my answer is that it depends on the "practitioner." As we all know, CBCT examinations are justified only when the benefits outweigh the risks. Here, the benefits from CBCT scan in orthodontic patients are quite different according to the practitioner. The ability and knowledge to use scan data varies from practitioner to practitioner. If he or she has little knowledge on the use of CBCT scan data (e.g., just see the volume image, and sometimes observe MPR image), a routine taking of CBCT in orthodontic cases might be blamed as unethical. 

**Figure 1:**
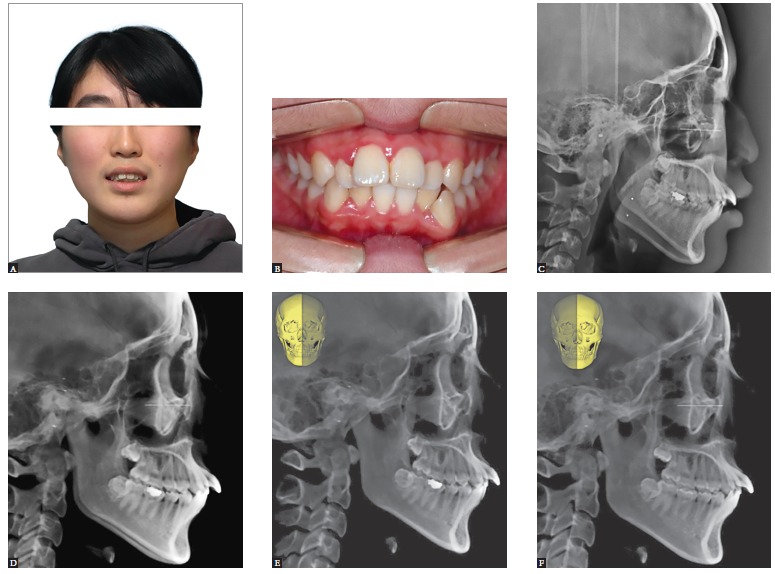
- Generation of cephalograms from cone-beam volume image. A 20-year-old lady came to us complaining of upper anterior protrusion (A and B). Cephalometric radiograph showed a significant difference in mandibular outline between the right and left side (C). Is this a real asymmetry? If so, which one is right or left? These questions cannot be answered with conventional lateral ceph, whereas they can be answered with cone-beam CT (CBCT). CBCT-generated cephalograms (CG Ceph) revealed no significant asymmetry (D). In order to differentiate right and left side clearly, right and left side half cephs were generated separately. It was revealed that the pattern of Class II high angle is more evident on the right side. Everything is clear with the help of half ceph generation (E and F).

On the other hand, it should be respected if a decision of CBCT taking was made by a practitioner based on the risk-benefit analysis. I routinely take CBCT in my orthodontic practice. It is because I am certain that the benefits always outweigh the risks in my practice. A number of images can be generated simply once one single scan of CBCT is obtained. As an example, all cephalograms are made on the basis of CBCT volume data.^4^ While cephalograms can be generated with perspective projection geometry for comparison with previous cephalograms taken with conventional cephalometer, cephalograms can be made also with parallel projection without any magnification and distortion. Accurate diagnosis is possible with CBCT-generated cephaolgrams (CG ceph). Uncertainty with perspective projection can be explained clearly with parallel projection of CG ceph. In addition, right side and left side can be generated separately, forming a unique image called 'half ceph.' Right and left difference can be evaluated clearly and simply by using a pair of half cephs (Fig 1). 

**Figure 2:**
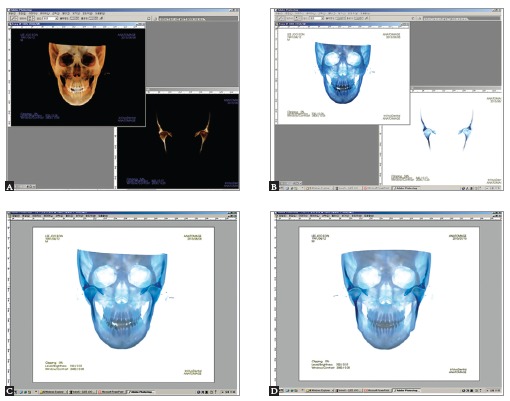
- Construction of composite cephalograms (composite ceph) from cone-beam volume image. In order to visualize condylar position in cephalograms, overall head and segmental view of the condyle area were captured in the 3D image program. The segmental view was created by removing overlapping area using the clipping and sculpt functions of the program (A). The captured images were imported into Photoshop, and color was inverted for visibility (B). Frontal cephalograms with highlighted condyles were generated by overlapping the two images, overall and segmental (C). After treatment, composite ceph can also be generated in the same way as in initial composite ceph (D). If we make an animated GIF using the two images, before and after composite cephs, treatment changes can be evaluated precisely and dynamically: this animated GIF (C and D) is available at http://goo.gl/sQ7R2B

We often want to see a structure (e.g., condyle) clearly in cephalograms. For this purpose, a segment can be generated from the volume image using the clipping and sculpt functions in the 3D image program. Overall and segmental images are overlapped in Photoshop program, generating a composite image with highlighted segment, condyle in this example. Using this image called 'composite ceph,' precise treatment evaluation has become possible in addition to an accurate diagnosis (Fig 2). 

However, it should be noted that this treatment evaluation during treatment is only possible when we have initial data. During the early stages of my 3D imaging, CT/CBCT was scanned in selected cases. Nevertheless, I had a difficulty with progress assessment during treatment of patients with no initial scan data. First, it happened in part of my patients, but it has become always the case with increasing ability on the use of CBCT scan data. 

**Figure 3:**
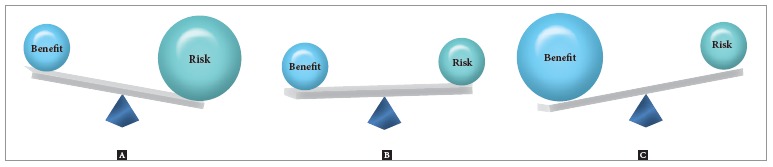
- Risk-benefit analysis on the use of CBCT in orthodontic practice. If a medical CT is used to obtain 3D image, the benefit usually does not outweigh the risk (A). However, the CBCT has reduced the risk considerably, so that the benefit may or may not outweigh the risk depending on the cases (B). With the development of computer technology, the benefit has outweighed the risk. However, this analysis might work only for practitioners who have a basic knowledge and ability to use CBCT scan data appropriately (C).

It is obvious that treatment quality is improved with the help of CBCT imaging. This is due to the fact that imaging is used not only for diagnosis, but also for progress evaluation during treatment. Unlike other dental treatments, progress evaluation during treatment is quite important in Orthodontics, and this is only possible with comparison with 'initial' data. There is an obvious trend that the benefits outweigh the risks (Fig 3).

## What are the great findings from your papers on craniofacial asymmetry that an orthodontist should keep in mind when analyzing a patient with this kind of disharmony? (Guilherme Thiesen)

My decision in diagnosis and treatment planning is based on the philosophy of minimally invasive Dentistry, and there is no exception for patients with facial asymmetry. As an example of chin deviation, how much deviation needs to be corrected? Based on menton deviation, two degrees or four degrees? All subjects with menton deviation over 4 degrees should be treated? Treatment decision needs to be based not only on the degree of asymmetry, but also on patient's perception of asymmetry. In some cases, patient's perception comes first. If asymmetry affects dental occlusion and oral health significantly, it should be corrected. However, if not, treatment decision needs to be based on patient's perception. 

For accurate diagnosis and treatment planning, more material is used in general. Considering the nature of asymmetry in the human body, more diagnostic material reveals more asymmetry. However, it should be noted that the use of more material is not for more treatment, it is for accurate diagnosis and understanding of the nature of asymmetry. This is really important in a patient with mild asymmetry. Some patients unduly worry about their asymmetry which is within normal limit. Just a verbal explanation such as "you do not need correction" does not work well in this kind of patient. If the patient does not understand the nature of his or her asymmetry, he or she might go and see other clinician, such as a plastic surgeon. For the management of this kind of patient, CBCT 3D analysis is crucial. The detailed explanation of the exact nature of their asymmetry could alleviate patients' concerns. It should be stressed that recently-developed 3D analysis is not for the practitioner, but for the patient. 

## You have developed, by cluster analyses, a classification system for facial asymmetries, admittedly a great challenge for clinical Orthodontics. This classification establishes four groups (based on menton deviation and ramal length differences) and advocates treatment protocols for each one of them. Do you think that every type of facial asymmetry can be included in one of these groups? Which group is the most prevalent? Which group presents the easiest and the toughest treatment for the clinician? (Guilherme Thiesen)

**Figure 4:**
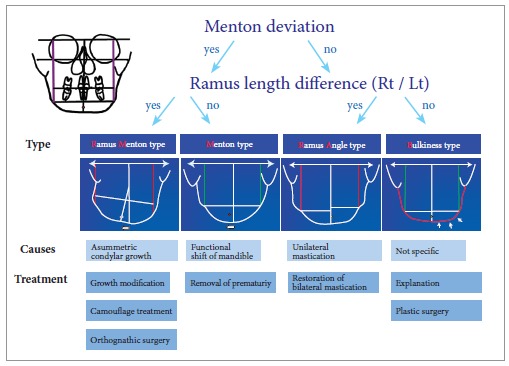
- Facial asymmetry can be classified into four groups: RM (Ramus Menton), M (Menton), RA (Ramus Angle), and B (Bulkiness). These groups are based on menton deviation and ramal length differences on frontal cephalograms. This classification allows the clinician to determine the cause of a given asymmetry and to formulate a proper treatment strategy for facial asymmetry patients.

Patients with facial asymmetry can be classified into four groups: RM, M, RA, and B types, as described in Figure 4.^5^^,^ 6 This classification is really useful in orthodontic practice because the causes of facial asymmetry can be identified easily. Moreover, the classification is possible just by using frontal cephalograms. Only two variables, menton deviation and ramus length differences, are needed. From this classification, accurate diagnosis can be made and a proper treatment plan can be established according to the type (Fig 4). 

Can all subjects with facial asymmetry be included in one of these groups? My answer is yes and no, more accurately 'yes' in orthodontic practice and 'no' for oral surgeon's practice which is dealing with craniofacial deformities. Nearly all asymmetry patients are classified into one of these four groups. On the other hand, it needs to be realized that all patients concerning asymmetry are not included into one of these groups. According to unpublished data, 91% of orthodontic patients who visited a university hospital and had frontal cephalograms taken for diagnostic purposes were classified into four groups. The remaining patients (9%) were diagnosed as being within normal limits.^5^

Considering the causes of each type of asymmetry, RM type is the toughest case in terms of treatment. The prognosis is questionable because the cause of this asymmetry is condylar growth difference between the right and left side. On the other hand, M type of asymmetry is the easiest case. Once any prematurity which may result in functional shift of mandible is removed, a balanced facial growth can be expected. While RM type is the toughest asymmetry, this is the most prevalent type of asymmetry in orthodontic clinic.^5^^,^ 6

## Do you indicate surgery-first orthognathic treatment for all your patients with marked skeletal disharmony in the three planes of space? (Telma Martins de Araújo)

The surgery-first (SF) approach demands more careful surgical planning and stronger collaboration between skilled orthodontists and surgeons in order to accurately predict post-surgical tooth movement and surgical movement. Therefore, previous advocates of this approach recommend only using the SF approach for mild to moderate skeletal discrepancies. However, the scope of this approach has been expanding with advances in 3D imaging technology and virtual orthodontic and surgical simulation.^7^

**Figure 5:**
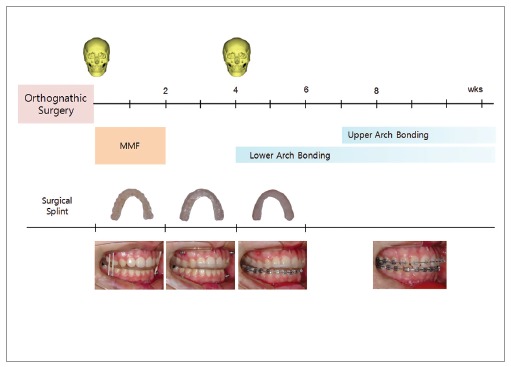
- Post-surgical management of unstable occlusion in surgery-first orthognathic treatment. In order to manage unstable occlusion, the surgical splint is left in the mouth after maxillomandibular fixation, so that the mandible is maintained in position even with mouth opening exercise. The acrylic resin wall can be added easily for retention of the splint. When brackets are placed on the opposite arch, the occlusal part of the splint is flattened in order to allow tooth movement of the opposite arch. With continued splint wear and stepwise modification, unwanted mandibular shifting can be prevented, indicating that more patients can benefit from surgery-first orthognathic treatment.

Another important issue is that unstable occlusion is inevitable after surgery in the SF approach. This could lead to surgical instability and interfere with subsequent orthodontic treatment. At the early stages of my SF practice, only selected cases with tripod or at least bilateral contact in the state of surgical occlusion were treated by using the SF approach. However, presently, unstable occlusion can be managed properly with the use of continued splint wear with stepwise modification, as illustrated in Figure 5. Unwanted mandibular shifting can be prevented by using surgical splint continuously. More and nearly all patients can benefit from the SF approach (Fig 5).

## In general, the advantage of surgery-first orthodontics is to avoid temporary deterioration in patient's appearance during the pre-surgical phase. In your experience, this is the key point of indication of this technique? What are the main difficulties that you have had in the finalization of these cases? (Maria Perpétua Mota Freitas)

It is certain that patients appreciate immediate improvement in facial appearance with the help of surgery-first (SF) orthodontics. However, the most important thing is that orthodontic tooth movement is easier and more physiologically favorable after surgical elimination of skeletal disharmony. It is because the direction of tooth movement for decompensation is not against soft tissue pressure. This is a benefit not only to the patient, but also to the practitioner. We have no difficulties even during the finalization phase. 

On the other hand, maintaining the condylar position during surgery is absolutely essential. In the conventional three-stage surgical orthodontic approach, minor changes in condylar position often go unnoticed because pre-surgical orthodontics enables stable occlusion to be routinely obtained after surgery. However, in the SF approach, even minor changes in the condylar position can cause unwanted shifting of the mandible due to the relatively unstable occlusion. Therefore, it is essential to monitor changes in condylar position before and after surgery. If a significant change is detected in post-surgery CBCT imaging, it is necessary to have the patient wearing a removable splint with continuous adjustment until stable occlusion is achieved and stable condylar position is obtained through the remodeling process.

## Considering the orthodontic phase after the surgery-first procedure, what would be your major concerns about the way patients face treatment? In other words, do you think the surgery-first criteria promote a different psychological impact from conventional orthodontic-surgical treatment, thus influencing patients' overall compliance (appointment non-attendance rate, hygiene, appliance breakage) and expectations? (Alexandre Trindade Simões da Motta)

The conventional three-stage surgical orthodontic approach, which includes pre-surgical orthodontics, surgery, and post-surgical orthodontics, has been well established as the gold standard in most cases. However, one of the drawbacks is the long pre-surgical treatment time that typically worsens facial appearance and exacerbates malocclusion. During the pre-surgical orthodontic period, all tooth movement is against soft tissue pressure. The teeth are leveled to a flat occlusal plane, relative to their own arches. Although the resulting occlusion facilitates proper positioning of the jaws, patients will experience discomfort throughout treatment of pre-surgical stage. Treatment does not improve quality of life, but rather deteriorates it, at least before surgery. In addition, patients become increasingly anxious about surgery under general anesthesia as the date of the surgery approaches. On the other hand, everything is completely the opposite in the surgery-first approach. I incorporated it into my practice in 2009. The last six years of experience have demonstrated greater patient satisfaction using the SF approach in surgical orthodontics. All of my patients appreciate treatment which is SF approach. Overall patients' expectations for SF orthodontics are beyond imagination, although their compliances are similar to those in conventional surgical orthodontics.

## Still on surgery-first orthodontics, how do you work with Spee curve in the treatment of asymmetries, knowing that this is a limiting factor for mandibular positioning during surgery? (Maria Perpétua Mota Freitas)

**Figure 6:**
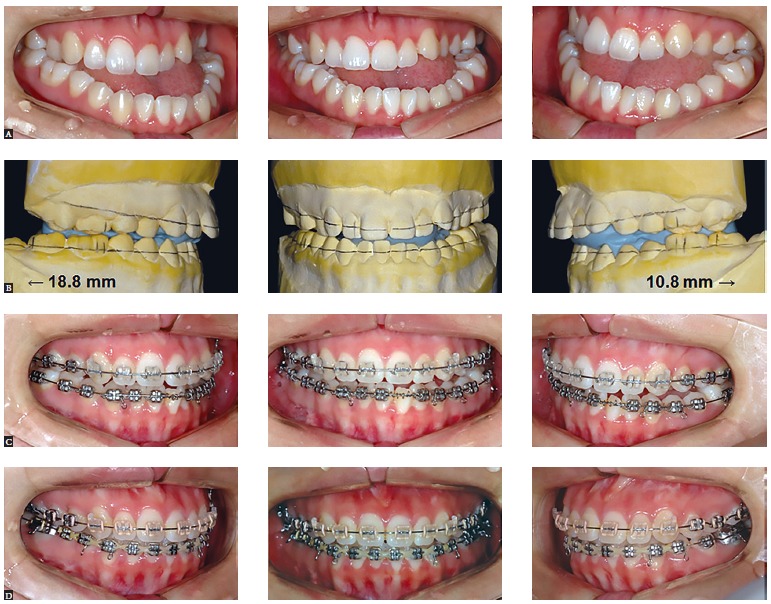
- A case example of skeletal Class III malocclusion with severe asymmetry treated with surgery-first approach. Although the patient showed a severe asymmetry and concomitant dental compensation in the transverse dimension, surgical occlusion could be obtained after simulation of post-surgical orthodontic tooth movement. After elimination of skeletal disharmony by two-jaw surgery - that included maxillary advancement and differential mandibular set-back using a surgeryfirst approach -, overall alignment and leveling by fixed orthodontic treatment was obtained so rapidly. Please note that Spee curve on the left side was relieved at the early stage of post-surgical orthodontic treatment. A, initial; B, surgical occlusion; C, two months into fixed treatment; D, seven months into fixed treatment.

In the surgery-first approach, occlusion cannot be used as a guide for surgical movement, and the surgeon is limited by tooth position in correcting skeletal discrepancy. This is really true in severe asymmetry cases which show deep Spee curve and dental compensation in the transverse dimension. Unlike surgeons, orthodontists can afford to simulate post-surgical orthodontic treatment. Accurate post-surgical tooth movement and surgical movement can be predicted, even in patients with severe skeletal discrepancy. Figure 6 shows an example of skeletal Class III with severe asymmetry treated by means of the SF approach. Although the patient showed severe asymmetry with deep Spee curve on one side, this did not act as a limiting factor for mandibular positioning during surgery. It is believed that nearly all patients can benefit from SF orthodontic treatment (Fig 6). 

## Recently, the use of mini-implants is becoming a routine in orthodontic treatment. This is also your routine, or do you have some restriction or specific indications for these accessories? (Maria Perpétua Mota Freitas)

Not any appliance can be used as a routine in orthodontic treatment, and mini-implants are no exception. It should be realized that mini-implant is not an orthodontic appliance, but merely an anchorage device. It is certain that there is a trend towards overuse of this device. Although the devices are very effective as a sure anchorage, they should be used only when necessary. If we overuse some devices habitually, we may misuse them. Indiscriminate use of this anchorage device can cause an imbalance in force system which can be a source of malpractice. 

## What is your experience with intrusion of posterior teeth using skeletal anchorage (mini-implants or mini-plates) to correct anterior open bite malocclusions in order to avoid orthognathic surgery? (Telma Martins de Araújo)

It is obvious that intrusion of posterior teeth using skeletal anchorage can be obtained to correct anterior open bite malocclusions. I also have many good cases treated with orthodontic miniscrew implants as anchorage. However, I do not recommend the use of skeletal anchorage in the correction of open bite to avoid orthognathic surgery. It is because stability cannot be guaranteed. When the patient refuses orthognathic surgery for some reasons, skeletal anchorage is used, but needs to be used as an adjunct to reduce anterior open bite.

## In the last AAO Congress held in San Francisco, USA, you gave a lecture on the use of the mini-tubes appliance (MTA) for alignment of anterior teeth. What are the indications and contraindications of this technique? What are the differences in using it in the buccal or in the lingual surfaces of anterior teeth? (Telma Martins de Araújo)

The mini-tube appliance (MTA), a round tube with diameter of 0.018-in and length of 3 mm, has been designed especially for anterior teeth alignment.^8^ It was originally developed to be used in young adults seeking a rapid improvement in their anterior esthetics. With the combined use of light NiTi wire and interproximal stripping, rapid alignment can be obtained within a very short period of time by using the MTA. Before the introduction of MTA, many patients with uneven anterior teeth received 'instant orthodontics' which is not orthodontic treatment, but ceramic veneer treatment by some of cosmetic dentists.^9^

**Figure 7:**
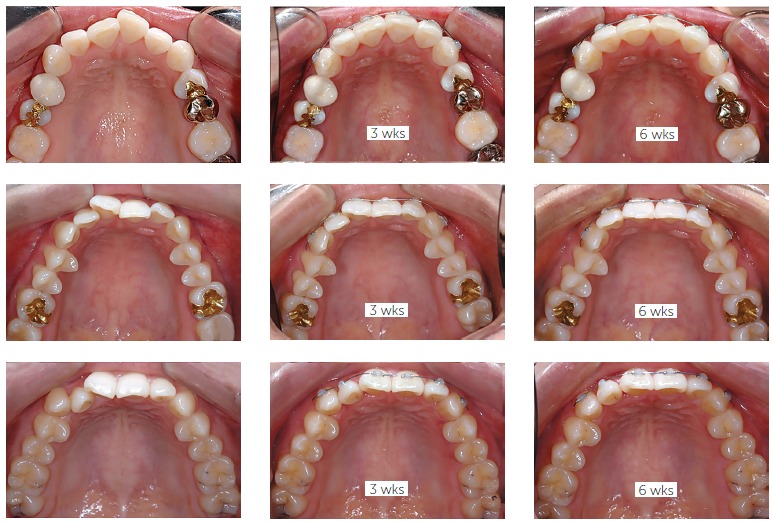
- A new appliance for rapid alignment. Mini-tube appliance (MTA), a round tube with diameter of 0.018-in and length of 3mm, has been especially designed for anterior teeth alignment. With the combined use of light NiTi wire and interproximal stripping, alignment can be obtained so rapidly. For this reason, MTA has been suggested as a sure alternative to ceramic veneers which require a considerable amount of tooth reduction.

While the MTA was used mostly in young adults during the early stages of MTA development, the scope of the MTA has expanded to all age groups of patients who need low-profile appliances and/or light force application. However, the appliances are used only in non-extraction cases because retraction of anterior teeth cannot be carried out by the MTA. On the other hand, some orthodontists prefer to use the MTA for initial alignment of anterior teeth even in extraction cases due to the characteristic of rapid alignment. It is interesting to note that anterior teeth are aligned with good gingival line leveling with the MTA, even without the use of rectangular wire (Fig 7). 

**Figure 8:**
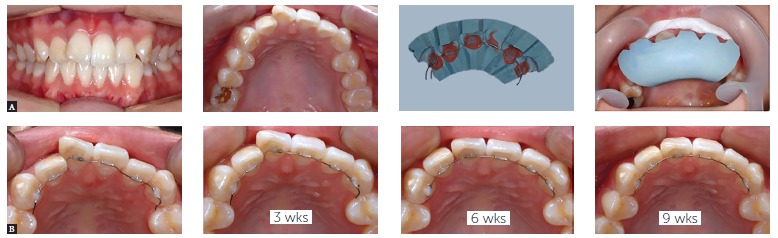
- Lingual application of mini-tube appliance (MTA). The mini-tubes can be placed also in the lingual surface. However, one potential problem for mini-tube is that a wire cannot be inserted easily into the tube, particularly in case of crowding. To overcome this limitation, a unique indirect bonding technique, named Indirect Bonding with Wire, has been developed. The tubes are attached to the models with an active wire, usually 012 NiTi. Not only the tubes, but also the wire can be transferred into the mouth using the indirect bonding tray. A) Indirect bonding; B) treatment progress.

With regards to labial or lingual application, it usually depends on the patient's demand. If a patient wants alignment with esthetic manner, the appliances are placed on the lingual surfaces. Sometimes, the MTAs are applied to lingual surfaces regardless of patient's desire. Also in this case, the appliances can be placed without any discomfort to the patient because the thickness of the appliance is very small, only 0.65 mm. One particular advantage of this unique appliance is that the appliance can be used as a retainer as it is after alignment, indicating no additional work for the retainer (Fig 8).^10^^,^ 11
